# Framework for headache management in pediatric patients with stroke and cerebrovascular lesions: A narrative review

**DOI:** 10.1111/head.70159

**Published:** 2026-08-11

**Authors:** Allison C. Hyland, Samuel J. Belfer, Christine K. Fox, Samantha L. Irwin, Rachel Vassar, Ann Oh, Sruthi Ilangovan, Amy A. Gelfand

**Affiliations:** ^1^ Department of Neurology and Weill Institute for Neuroscience University of California San Francisco San Francisco California USA; ^2^ Department of Neurology, Division of Neurocritical Care Children's Hospital of Philadelphia, University of Pennsylvania Philadelphia Pennsylvania USA; ^3^ Department of Pediatrics University of Pennsylvania Perelman School of Medicine, Children's Hospital of Philadelphia Philadelphia Pennsylvania USA; ^4^ Department of Neurology University of Pennsylvania Perelman School of Medicine, Children's Hospital of Philadelphia Philadelphia Pennsylvania USA; ^5^ Child & Adolescent Headache Program University of California San Francisco San Francisco California USA; ^6^ Department of Neurology Dell Medical School at the University of Texas at Austin Austin Texas USA; ^7^ UT Health Austin Pediatric Neurosciences at Dell Children's Dell Children's Medical Center and UT Health Austin Austin Texas USA

**Keywords:** chronic headache, headache management, headache prevention, headache treatment, pediatric headache, stroke in the young

## Abstract

**Objective:**

To present a practical, evidence‐based framework for the management of headache disorders in pediatric patients with prior stroke or underlying cerebral vascular lesions, with particular attention to safety and efficacy of pharmacologic and non‐pharmacologic therapies.

**Background:**

Chronic headache is frequent after ischemic or hemorrhagic stroke (reported in 12–57% of patients) and is also common in cerebrovascular disorders. Pediatric patients with these conditions face unique therapeutic challenges because many typical headache medications have vasoactive or blood pressure–modulating effects that may increase cerebrovascular risk.

**Methods:**

We performed a comprehensive MEDLINE literature review (July 2024) searching combinations of headache medication classes paired with specific neurovascular conditions. Inclusion criteria: English or translated articles since 1990, comprising human and animal in vitro/in vivo studies, narrative reviews, case series/reports. Extracted data were organized by vascular condition, with summary tables of preventive and acute interventions categorized by evidence of efficacy, safety, and theoretical risk.

**Results:**

Across conditions, vasoconstrictive agents (triptans, ergots) remain generally contraindicated or used with extreme caution in patients with vascular lesions due to risk of ischemia or hemorrhage. Anti‐hypertensive agents (β‐blockers, calcium channel blockers [CCBs], angiotensin II receptor blockers [ARBs]) are generally safe and may confer additional vascular benefit in post‐stroke headache. Calcitonin gene‐related peptide (CGRP) ‐targeting therapies are not formally contraindicated but may impair compensatory vasodilation in flow‐dependent vascular lesions. Anti‐seizure medications, antidepressants, melatonin, and non‐pharmacologic therapies appear to have favorable risk–benefit profiles, although direct data in these populations are limited. Interventions addressing underlying vascular lesions (e.g., arteriovenous fistulas [AVF] embolization, arteriovenous malformations [AVM] or cavernoma resection, pial synangiosis) often lead to headache improvement.

**Conclusion:**

In pediatric patients with cerebrovascular disease who have headaches, individualized management is essential. Drug selection must incorporate vascular pathophysiology, hemodynamic stability, and collateral flow considerations. More prospective, pediatric‐specific and lesion‐specific studies are needed to guide optimal headache therapies in this complex population.

## Plain Language Summary

Many young people continue to have persistent or recurring headaches after a stroke or because of underlying blood vessel problems in the brain, yet standard headache treatments may carry extra risks in this setting. We reviewed available evidence and found that certain commonly used headache medications (such as vessel constrictors) may be unsafe in these patients, while others (such as blood pressure drugs, seizure‐prevention agents, or non‐drug therapies) appear safer and may be tailored to their specific vascular condition. The results of this study can support doctors and patients in making better, individualized choices about headache care when stroke or cerebrovascular disease is present.

**Abbreviations**
ACA: anterior cerebral artery
ACE: angiotensin‐converting enzyme
AChE: acetylcholinesterase
ARBs: angiotensin II receptor blockers
ASMs: anti‐seizure medications
AVFs: arteriovenous fistulas
AVMs: arteriovenous malformations
BBs: beta‐blockers
BP: blood pressure
CADASIL: cerebral autosomal dominant arteriopathy with subcortical infarcts and leukoencephalopathy
CBT: cognitive behavioral therapy
CCBs: calcium channel blockers
CCFs: carotid cavernous fistula
CCMs: cerebral cavernous malformations
CGRP: calcitonin gene‐related peptide
CNS: central nervous system
DHE: dihydroergotamine
FDA: United States Food and Drug Administration
ICA: internal carotid artery
mAbs: monoclonal antibodies
MCA: middle cerebral artery
MELAS: Mitochondrial Encephalomyopathy, Lactic Acidosis and Stroke‐like Episodes Syndrome
MM: Moyamoya vasculopathy
MMD: Moyamoya disease
MMS: Moyamoya syndrome
NMDA: N‐methyl‐D‐aspartate
NSAIDS: non‐steroidal anti‐inflammatory drugs
PI: principal investigator
PRES: posterior reversible encephalopathy syndrome
RCVS: reversible cerebral vasoconstriction syndrome
SNRIs: serotonin‐norepinephrine reuptake inhibitors
SWS: Sturge–Weber syndrome
TCAs: tricyclic antidepressants
VBI: vertebrobasilar insufficiency
VPA: valproic acid

## BACKGROUND

Chronic headache is a frequent and likely underrecognized consequence of both ischemic and hemorrhagic stroke, affecting 12–57% of patients across etiologies.^1^, ^2^, ^3^, ^4^, ^5^ Beyond acute cerebrovascular events, headache is also common in individuals with underlying vascular disorders such as arteriovenous malformations (AVMs), dural arteriovenous fistulas (AVFs), cerebral cavernous malformations (CCMs), cerebral autosomal dominant arteriopathy with subcortical infarcts and leukoencephalopathy (CADASIL), mitochondrial encephalomyopathy, lactic acidosis, and stroke‐like episodes (MELAS), moyamoya vasculopathy (MM), reversible cerebral vasoconstriction syndrome (RCVS), Sturge–Weber syndrome (SWS), and vascular stenosis or insufficiency.^6^, ^7^ In these conditions, headache may arise from altered cerebral perfusion, impaired autoregulation, endothelial dysfunction, or recurrent microvascular injury, creating a clinical scenario with distinct considerations from other headache disorders.^8^, ^9^, ^10^

Management of headache in patients with elevated cerebrovascular risk is uniquely challenging. Many of the most effective acute and preventive headache therapies—including triptans, ergots, beta‐blockers (BBs), calcium‐channel blockers (CCBs), angiotensin II receptor blockers (ARBs), tricyclic antidepressants (TCAs), and serotonin‐norepinephrine reuptake inhibitors (SNRIs)—have vasoactive or hemodynamic properties that may exacerbate ischemia, impair collateral circulation, or provoke hemorrhage in susceptible patients.^11^, ^12^, ^13^, ^14^, ^15^ Newer calcitonin gene‐related peptide (CGRP)–targeting medications introduce additional complexity: monoclonal antibodies have long half‐lives and limited central nervous system (CNS) penetration, whereas small‐molecule gepants can cross the blood–brain barrier and may interfere with compensatory vasodilation during acute ischemia.^16^, ^17^, ^18^, ^19^ These mechanistic considerations are critical when treating patients who rely on fragile collaterals or altered vascular pathways for cerebral perfusion.

Despite the pediatric focus of this review, pediatric‐specific data regarding headache management in the context of cerebrovascular disease remain extremely limited. Much of the available literature describing medication safety, efficacy, and adverse effects in these conditions derives from adult populations, often including young adults whose physiology may partially overlap with adolescents. As a result, many therapeutic considerations discussed herein are extrapolated from adult studies rather than derived from pediatric clinical trials. Consequently, the management of headache in children and adolescents with cerebrovascular lesions is not standardized, reflecting both the rarity and heterogeneity of these disorders as well as the absence of robust prospective studies. Comprehensive pediatric investigations are needed to more clearly establish the safety and efficacy of commonly used headache therapies in these patients. Until such data are available, our aim was to synthesize existing evidence where possible and to incorporate pathophysiologic rationale where clinical data are lacking, providing clinicians with a practical framework to guide management of headache in children with these rare but clinically important cerebrovascular conditions.

## METHODS

### Literature search strategy and selection criteria

Comprehensive searches in MEDLINE were conducted in July 2024. Topic selection and definition of the scope of the narrative review were performed by A.C.H., C.K.F., and A.A.G. Search terms paired classes of headache medication with specific neurovascular conditions (e.g. “Calcium Channel Blocker” AND “Ischemic Stroke;” “Triptan” AND “Reversible Cerebral Vasoconstriction Syndrome”). Classes of headache medications included agents commonly used for acute treatment (e.g., triptans, ergots, non‐steroidal anti‐inflammatory drugs [NSAIDs], anesthetics) and preventive treatment (e.g., BBs, CCBs, angiotensin‐converting enzyme [ACE] inhibitors, ARBs, anti‐seizure medications [ASMs], tricyclic antidepressants [TCAs], serotonin‐norepinephrine reuptake inhibitors [SNRIs], calcitonin gene‐related peptide [CGRP] monoclonal antibodies and gepants, and onabotulinum toxin). Neurovascular conditions included the broad categories of hemorrhagic and ischemic stroke, as well as specific syndromes: AVF, AVM, CCM, CADASIL, MELAS, MM, RCVS, SWS, and vascular insufficiency/stenosis. Studies including children and adolescents were prioritized whenever available. However, given the limited pediatric‐specific literature addressing headache management in the context of cerebrovascular disease, evidence from adult populations was included when pediatric data were unavailable.

### Eligibility Criteria

Inclusion criteria were defined as studies in the English language or with translation available and with publication dates after 1990. Sources included in vitro animal and human studies, in vivo animal and human studies, systemic and narrative reviews, and case studies and series. Duplicates were removed.

### Data collection

Data extraction from articles, tables, and figures for eligible literature was performed independently by authors (A.C.H., C.K.F., S.L.I., R.V., A.O.). Extracted data was then organized by neurovascular condition, and initial drafts were submitted and reviewed for clarity by A.C.H. and S.J.B. Tables 1 and 2 outline preventive and acute interventions respectively, categorized by efficacy, safety, and evidence‐based and/or theoretical risk and were composed by A.C.H. and S.J.B. after synthesis of the manuscript and review of sources.

**TABLE 1:** Acute headache interventions in patients with a history of vascular disorders. [Color table can be viewed at wileyonlinelibrary.com]

Neurovascular condition	No direct evidence, but theoretical benefit	Evidence suggests efficacy but risk present	Evidence suggests ineffective or high risk or no direct evidence, but theoretical risk
Ischemic stroke		Triptans^20^, ^21^, ^22^ DHE^22^ CGRP mAbs^17^, ^23^, ^24^, ^25^, ^26^, ^27^, ^28^	Gepants^17^, ^23^, ^25^, ^26^, ^28^, ^29^
Hemorrhagic stroke		Triptans^20^, ^21^, ^30^ DHE^30^ CGRP mAbs^17^, ^24^, ^27^	Gepants^17^, ^24^
*Congenital or acquired vascular malformations*			
AVM		Triptans^31^, ^32^ DHE^31^ CGRP mAbs* Gepants*	
AVF		Triptans^31^, ^32^, ^33^ DHE^31^ CGRP mAbs* Gepants*	
CCM	NSAIDs, ASA^34^, ^35^, ^36^ Triptans^34^		
*Genetic vasculopathies*			
CADASIL		Triptans^37^	CGRP mAbs^24^
MELAS	Triptans^38^, ^39^		
SWS		NSAIDs, ASA^40^ Triptans^40^, ^41^, ^42^ DHE* CGRP mAbs* Gepants*	
*Acquired or progressive vasculopathies*			
MM Vasculopathy	NSAIDs, ASA^43^	Triptans^44^, ^45^, ^46^ DHE^44^, ^45^, ^46^	CGRP mAbs^17^, ^24^ Gepants^17^, ^24^, ^29^
RCVS		CGRP mAbs^17^, ^24^ NSAIDs^47^	Gepants^17^, ^24^, ^29^, ^48^ DHE^47^, ^49^, ^50^, ^51^, ^52^, ^53^ Triptans^47^, ^49^, ^50^, ^51^, ^52^, ^53^, ^54^ Glucocorticoids^55^
Other vascular insufficiency/stenosis		Triptans^56^, ^57^, ^58^, ^59^, ^60^, ^61^ DHE^56^, ^57^ CGRP mAbs*	Gepants*

*Note*: Evidence for benefit and risk levels denoted by references after each medication. Medications with only theoretical benefit based on pathophysiology denoted with *. Green indicates “no direct evidence, but theoretical benefit” yellow indicates “evidence suggests efficacy but risk present”; and pink/red indicated “evidence suggests ineffective, high risk, or no direct benefit but theoretical risk”.

Abbreviations: ASA, acetylsalicylic acid (aspirin); AVF, arteriovenous fistula; AVM, arteriovenous malformation; CADASIL, cerebral autosomal dominant arteriopathy with subcortical infarcts and leukoencephalopathy; CCM, cerebral cavernous malformation; CGRP mAbs, calcitonin gene‐related peptide monoclonal antibodies; DHE, dihydroergotamine; MELAS, mitochondrial encephalomyopathy, lactic acidosis, and stroke‐like episodes; MM, Moyamoya; NSAID, non‐steroidal anti‐inflammatory drug; RCVS, reversible cerebral vasoconstriction syndrome; SWS, Sturge–Weber syndrome.

**TABLE 2:** Preventative headache interventions in patients with a history of vascular disorders. [Color table can be viewed at wileyonlinelibrary.com]

Neurovascular condition	No direct evidence, but theoretical benefit	Evidence suggests efficacy but risk present	Evidence suggests ineffective or high risk or No direct evidence, but theoretical risk
Ischemic stroke	ARBs BBs CCBs *Others*: Lamotrigine^62^	CGRP mAbs^17^, ^23^, ^24^, ^25^, ^26^, ^27^, ^28^	Gepants^17^, ^23^, ^25^, ^26^, ^28^, ^29^ *Others*: Pregabalin^63^ Carbamazepine^64^ Levetiracetam^64^
Hemorrhagic stroke	ARBs^13^, ^14^, ^65^, ^66^, ^67^, ^68^, ^69^ BBs^14^, ^65^ CCBs^65^ *Others*: Lamotrigine,^62^ Melatonin^70^, ^71^, ^72^	CGRP mAbs^17^, ^24^, ^27^	Gepants^17^, ^24^
*Congenital or acquired vascular malformations*			
AVM	ARBs^73^ *Others*: Surgical Intervention^74^, ^75^, ^76^, ^77^	CGRP mAbs*	Gepants*
AVF	ARBs^78^ *Others*: Surgical Intervention^33^, ^79^, ^80^, ^81^	CGRP mAbs*	Gepants*
CCM	BBs		
*Genetic vasculopathies*			
CADASIL	ARBs^82^ BBs^83^ CCBs^84^, ^85^	CGRP mAbs*	Gepants*
MELAS	BBs^86^ CCBs^38^, ^87^	CGRP mAbs^38^, ^87^	Gepants*
SWS	CCBs^88^ *Others*: Lamotrigine^41^	CGRP mAbs*	Gepants* *Others*: Topiramate* TCAs*
*Acquired or progressive vasculopathies*			
MM vasculopathy		ARBs* BBs^89^ CCBs^89^, ^90^, ^91^	CGRP mAbs^17^, ^24^ Gepants*
RCVS	BBs^54^, ^92^ CCBs^48^, ^54^, ^92^, ^93^, ^94^		CGRP mAbs* Gepants*
Other vascular insufficiency/stenosis		ARBs^95^ BBs^96^ CCBs^93^	CGRP mAbs* Gepants*

*Note*: Evidence for benefit and risk levels denoted by references after each medication. Medications with only theoretical benefit based on pathophysiology denoted with *. Green indicates “no direct evidence, but theoretical benefit” yellow indicates “evidence suggests efficacy but risk present”; and pink/red indicated “evidence suggests ineffective, high risk, or no direct benefit but theoretical risk”.

Abbreviations: ARB, angiotensin receptor blocker; AVF, arteriovenous fistula; AVM, arteriovenous malformation; BB, beta blocker; CADASIL, cerebral autosomal dominant arteriopathy with subcortical infarcts and leukoencephalopathy; CCB, calcium channel blocker; CCM, cerebral cavernous malformation; CGRP mAbs, calcitonin gene‐related peptide monoclonal antibodies; MELAS, mitochondrial encephalomyopathy, lactic acidosis, and stroke‐like episodes; MM, Moyamoya; RCVS, reversible cerebral vasoconstriction syndrome; SWS, Sturge–Weber syndrome; TCA, tricyclic antidepressant.

## RESULTS

A summary of therapies discussed in this review is provided in Tables 1 and 2, which categorize commonly used headache treatments according to *acute and preventive indications*, along with available evidence regarding safety and theoretical risks in patients with cerebrovascular disorders.

## ISCHEMIC STROKE

Headache is common in acute ischemic stroke, seen in 55–100% of patients with extracranial cervical artery dissection, 15–41% of patients with larger artery atherosclerosis, 13–33% with small vessel disease, and 9–39% with cardioembolic strokes.^97^ Up to 44% of patients with posterior circulation stroke have associated headache, with female sex being a risk factor for headache development.^5^ Headache beyond the acute period is also common, with persistent headache longer than 3 months reported in 12% of patients.^4^ Persistent headache is more likely in younger individuals, females, and those with pre‐existing headache disorders or comorbid post‐stroke fatigue or depression.^98^ Headaches were more commonly reported as tension‐type headache than migraine.^5^, ^97^, ^98^

### Triptans and ergots

Triptans are serotonin 1B/1D/1F agonists that are increasingly used as first‐line therapy for acute migraine attacks,^99^ and several triptans are approved for use in the pediatric population.^100^, ^101^, ^102^, ^103^ The mechanism of action of triptans in the treatment of migraine has not been clearly identified. Although there are minimal vasoconstrictor effects on intracranial arteries in vivo during migraine,^104^ triptans are generally contraindicated in patients with a history of ischemic stroke due to theoretical risk.^97^, ^105^, ^106^ This risk may be especially significant in patients with more cardiovascular risk factors.^11^ Dihydroergotamine (DHE) is the most used ergot‐derivative for headache. DHE has less selective receptor binding and slower diffusion from its receptors, which leads to longer‐lasting effects and more side effects than most triptans.^107^, ^108^, ^109^ DHE is also contraindicated in patients with a history of ischemic stroke due to theoretical risk related to vasoactive effects.^97^ However, there may be more nuance in the mechanism of action of these medications for headache after ischemic stroke. In a mouse model, DHE significantly decreased cerebral blood flow, while eletriptan had no effect,^22^ indicating potential for heterogenous intracranial effects.

### Anti‐hypertensive agents

Anti‐hypertensive agents such as BBs, CCBs, and ARBs are commonly used to address risk factors for ischemic stroke in adults and may be considered to address headache in patients with comorbid hypertension^15^, ^110^, ^111^, ^112^, ^113^, ^114^, ^115^, ^116^; however, their use for headache management after stroke is not specifically established in guidelines or supported by dedicated clinical trials.

### CGRP‐targeting medications

CGRP is released from trigeminal nerve endings during migraine attacks and contributes to the activation and sensitization of the trigeminovascular system. CGRP monoclonal antibodies (mAbs) prevent migraine by selectively binding to CGRP or its receptor, thereby blocking biological activity without crossing the blood–brain barrier in substantial concentrations.^117^ Fremanezumab was recently approved for preventative treatment of episodic migraine in pediatric patients aged 6–17 years who weigh greater than 45kg.^118^ CGRP medications are not contraindicated at present in patients with a history of ischemic stroke, but these agents have theoretical risk given potent vasodilatory properties of CGRP.^23^, ^105^ In mouse models, gepants (olcegepant and rimegepant) increased ischemic bed size, decreased collateral flow, reduced reperfusion success, and worsened neurological outcomes during middle cerebral artery (MCA) occlusion.^29^ These medications should be used with caution in patients with ischemic stroke.

### Other agents

NSAIDs are not explicitly contraindicated in ischemic stroke‐associated headaches. Chronic NSAID use has been shown to increase the risk of ischemic stroke,^119^ but increased rates of ischemic stroke in individuals with migraine cannot be explained by NSAID use alone.^120^ Other pharmacological agents have potential benefit for central post‐stroke pain including headaches, but there is a lack of specific evidence for use in headaches secondary to ischemic stroke. One randomized trial of pregabalin use in post‐stroke pain did not find significant improvement in headache frequency or severity.^63^ Similarly, carbamazepine and levetiracetam were not clearly beneficial based on small, randomized trials.^64^ One placebo‐controlled double‐crossover trial of lamotrigine in 30 patients with post‐stroke pain found significant reduction in pain scores at 200 mg/day in adults, but not at lower doses,^62^ potentially suggesting some benefit for post‐stroke headache. Non‐pharmacological methods (e.g., cognitive behavioral therapy [CBT], biofeedback, physical therapy, non‐invasive brain stimulation) have shown evidence of efficacy in pediatric headache,^121^, ^122^, ^123^, ^124^, ^125^ and are also suggested to be safe and well‐tolerated in headache after ischemic stroke.^97^, ^106^ OnabotulinumtoxinA is efficacious for preventative management of chronic migraine^126^, ^127^ in adults, but no specific data is available in pediatric headache post‐ischemic stroke.

## HEMORRHAGIC STROKE

### Triptans and ergots

Ergot‐derivatives and triptans are contraindicated in patients with a history of hemorrhagic stroke due to theoretical bleeding risk.^97^ Observational studies have linked patients with triptan prescriptions to hemorrhagic stroke, but the risk of stroke was found to be a consequence of underlying migraine, not triptan use.^128^ Additionally, case reports have tied overuse of these medications to bilateral intraparenchymal hemorrhages.^129^

### Anti‐hypertensive agents

Despite theoretical concerns in patients with flow‐dependent neurovascular lesions, the literature suggests that BBs, ACEs, and ARBs following hemorrhagic stroke are safe and may improve long‐term stroke outcomes.^13^, ^14^, ^15^, ^65^, ^66^, ^97^, ^130^, ^131^ As in ischemic stroke, there are no dedicated studies demonstrating potency of anti‐hypertensive agents in post‐hemorrhagic stroke headache reduction. Due to favorable safety profile, limited side effects, and efficacy in migraine, anti‐hypertensive agents remain an option for headache prevention; however, when utilized, care should be taken to avoid a rapid reduction in blood pressures upon initiation.

### CGRP‐targeting medications

Data on CGRP agents in hemorrhagic stroke are limited. These medications are not directly contraindicated in patients with history of stroke, but based on their mechanism they could theoretically limit compensatory vasodilation after stroke and may pose a risk to patients with intracranial hemorrhage.^106^ Blocking CGRP poses a theoretical risk impairment of compensatory cerebrovascular responses, especially in patients with flow‐dependent or collateral‐dependent lesions.^16^, ^17^, ^24^ Additionally, erenumab, a monoclonal antibody targeting the CGRP receptor, may increase blood pressure, which could be problematic in patients with a history of hypertensive intracranial hemorrhage.^25^, ^26^, ^27^

### Other agents

Medications with lower vasoactive effects—such as TCAs, SNRIs, CCBs, α2δ ligands (e.g., gabapentin, pregabalin), ASMs (e.g. carbamazepine, levetiracetam, lamotrigine), N‐methyl‐D‐aspartate (NMDA) antagonists (e.g., ketamine, amantadine, memantine), and intravenous lidocaine and steroids—are recommended.^106^ Lamotrigine has moderate evidence supporting its efficacy in treating headache after hemorrhagic stroke.^62^ Non‐pharmacological methods (e.g., repetitive transcranial magnetic stimulation, transcranial direct current stimulation, CBT, physical therapy, biofeedback) are also suggested for treatment of headache, presumably with little risk.^106^ Similarly, the use of botulinum toxin has been efficacious for primary headache disorders,^132^, ^133^ and is not contraindicated in hemorrhagic stroke, but is without specific evidence in this condition. Peripheral nerve block has been shown to reduce refractory headache pain scores in adults with subarachnoid hemorrhage in a small pilot study^134^; however, further investigation in larger cohorts is needed, and pediatric‐specific data is currently lacking.

Melatonin, used for migraine prevention, has a favorable side effect profile.^135^ One study proposed melatonin as a promising neuroprotective candidate for its function as an antioxidant, anti‐apoptotic agent, anti‐inflammatory agent, and a potential mediator of post‐hemorrhagic parenchymal injury.^70^ In a rat model of hemorrhagic stroke, melatonin ameliorated secondary brain injury in intracranial hemorrhage by impacting apoptosis, inflammation, oxidative stress, DNA damage, brain edema, and blood–brain‐barrier injury^71^; however, retrospective matched‐cohort study found no mortality benefit nor adverse effects in patients with aneurysmal subarachnoid hemorrhage receiving melatonin, but effect on headache was not evaluated.^72^ Melatonin might be helpful for headache prophylaxis while also minimizing long‐term injury after intracranial hemorrhage, and favorable side effect profile merits trial in patients with post‐hemorrhagic stroke and headache.

## CONGENITAL OR ACQUIRED VASCULAR MALFORMATIONS

### AVMs

AVMs are cerebrovascular lesions characterized by direct arteriovenous shunting of blood via a tangle of abnormal vessels instead of a normal intervening capillary bed.^74^ AVMs can present with migraine‐like headaches and contralateral visual aura.^136^ The mechanism of headache attributed to AVM is unknown, with increased intracranial pressure, steal phenomenon, and cortical spreading depression as proposed mechanisms.^74^ Intranidal aneurysms can be present within AVMs, which are an independent risk factor for hemorrhage, but their role in headache has not been explored.^137^ While acute vasoactive agents are generally avoided, several preventive treatments are not directly contraindicated in patients with AVM.

#### Surgical interventions

Surgical intervention can achieve symptom resolution in headache attributed to AVM. Gamma knife stereotactic radiosurgery reduced headache severity by 44% and headache frequency by 53% in a retrospective analysis.^77^ Several surgical case series demonstrate the safety and efficacy of using embolization, radiosurgery, microsurgical resection, or a multimodal approach to successfully treat headache.^74^, ^75^ One recent review demonstrated surgical intervention to be superior for AVM‐related headache compared to conservative treatment.^76^

#### Triptans and ergots

In vitro, both DHE and sumatriptan induced significant contraction in the middle meningeal artery, with DHE also showing venous contractile properties.^31^ There is complementary human in vivo evidence of vasoconstriction from direct sumatriptan injection into normal arteries, but not hypertrophied dural vessels feeding an AVM.^32^ This suggests AVMs with significant shunting and feeder hypertrophy may have reduced risk of vasoreactivity to triptans.^32^

#### Anti‐hypertensive agents

Anti‐hypertensives are leveraged for treatment of AVMs, as they may decrease the risk of intranidal aneurysm rupture^73^; however, there is no specific data on anti‐hypertensive use on the frequency or severity of headaches.

### AVFs

AVFs are abnormal communications between meningeal arteries and dural venous sinuses and/or cortical veins.^138^ They are distinct from AVMs in that there is no interluding tangle of vessels between inappropriately shunted arterial‐to‐venous blood.^139^ Dural AVFs (dAVFs), a subtype of AVMs located in the dura mater or arachnoid layer, account for 10–15% of all intracranial AVMs.^140^ They result from abnormal shunting of high‐pressure blood directly into low‐pressure systems (e.g. dural sinus vein, cortical and meningeal veins), and can be acquired in association with dural venous sinus thrombosis, venous hypertension, previous craniotomy, and head trauma.^140^, ^141^ In a study of 40 patients, carotid cavernous fistula (CCFs) presented with migraine‐like headache, while other dAVF subtypes presented with non‐migraine‐like headache ipsilateral to the fistula.^79^ Retro‐orbital pain and ocular symptoms have been identified as key symptoms suggestive of CCFs due to venous congestion of the superior ophthalmic vein.^79^, ^141^

#### Surgical interventions

Treatment of chronic headache in patients with AVF should prioritize addressing the underlying vascular lesion, with endovascular therapy recommended as first‐line for most intracranial dAVFs and surgery reserved for select cases.^142^ Embolization of the fistula is the most common successful intervention for headaches attributed to dAVF.^33^, ^79^, ^80^ Endovascular treatment of a pulsatile scalp AVF in a 6‐year‐old child with Hartnup disease led to the complete resolution of recurrent right‐sided migrainous headaches.^81^ There are also several case reports of migraine‐like headaches and ocular symptoms abating following embolization of AVFs.^33^, ^79^, ^143^, ^144^ For patients whose headaches persist after AVF treatment or in whom intervention is not indicated, pharmacologic therapies should be considered.^145^

#### Triptans and ergots

Vasoconstrictive agents (e.g., triptans, ergots) are generally avoided due to theoretical risk of AVF rupture, although robust data are lacking. More data is needed to determine efficacy and safety of these medications.

#### Anti‐hypertensive agents

Anti‐hypertensive agents have not been associated with increased risk of hemorrhagic presentation in patients with AVF,^15^, ^130^, ^131^, ^145^ and acetylcholinesterase (AChE) inhibitors may even confer a lower risk of hemorrhagic events.^146^ In patients with AVF, these agents may also correct neurohormonal imbalances associated with large AVFs and high‐output states,^147^ findings supported by animal models^78^, ^148^, ^149^; however, there is no direct evidence supporting efficacy for headache relief in this population. Careful monitoring for hypotension or renal effects is advised when used in this population.

### CCMs

CCMs are clusters of abnormal, thin‐walled blood vessels in the brain or spinal cord, with a prevalence of 0.5–1%.^150^, ^151^ Patients are frequently asymptomatic, but some present with seizures, focal neurologic deficits, and/or headaches.^152^, ^153^, ^154^ CCMs are also estimated to bleed at a rate of 2% annually, with hemorrhagic episodes clustered over time.^154^

#### Triptans and ergots

Data is limited, but vasoconstricting medications are theoretically unlikely to affect CCMs due to the lack of smooth muscle within these lesions. This is supported by a prospective study which found no association between triptan use and hemorrhage risk.^34^ Therefore, triptans and ergots can be used in patients with CCMs.

#### Anti‐hypertensive agents

Anti‐hypertensive agents are considered safe in patients with CCMs and are increasingly used to reduce hemorrhage risk and lesion size.^155^, ^156^, ^157^, ^158^, ^159^, ^160^, ^161^ Evidence for efficacy in prevention or treatment of headache was not found.

#### Other agents

NSAIDs have traditionally been avoided in people with CCMs due to their antithrombotic effects theoretically increasing bleeding risk^162^; however, recent studies failed to demonstrate increased hemorrhage risk with NSAID use.^34^, ^35^ Additionally, a recent meta‐analysis found antithrombotic therapy was associated with lower intracranial hemorrhage risk.^36^ These data suggest that the theoretical risk of hemorrhage may not be realized in vivo, but these medications should be used with caution until additional data is available.

## GENETIC VASCULOPATHIES

### CADASIL

CADASIL is a genetic condition caused by pathogenic variants in *NOTCH3*, manifesting clinically as stroke, migraine (often with aura), and other vascular complications.^37^ Fifty‐five to 75% of cases of CADASIL feature migraine, and in greater than 40% of cases, migraine with aura was the inaugural symptom.^163^

#### Triptans and ergots

Triptans are generally contraindicated in patients with CADASIL due to risk of vasoconstriction via activation of 5HT‐1B and ‐1D receptors; however, newer data suggest triptans can be an effective acute treatment with no complications in 46% of patients with CADASIL,^37^ indicating it may be safe for some patients.

#### Anti‐hypertensives

Blood pressure management is central to mitigating sequelae of CADASIL, including migraine. Case reports suggest uncontrolled hypertension as a modifiable risk factor for severity of symptoms.^83^ BBs, CCBs, and ARBs are used for headache prevention in CADASIL.^37^ Thirty‐two percent of patients with CADASIL receiving BBs for migraine reported benefit.^37^ CCBs verapamil and lomerizine have both shown potential in reducing headache burden and stroke risk in this population.^82^, ^84^, ^85^, ^164^ ARB use with a low‐protein diet may offer benefit for headache and kidney health in patients with CADASIL with renal disease.^82^

### MELAS

MELAS is a mitochondrial disorder most associated with a mutation in the *MT‐TL1* gene, but represents a heterogenous group of mitochondrial disorders.^165^ This syndrome can present with headaches associated with nausea and vomiting, and can be difficult to treat.^165^ While the exact mechanism of stroke‐like episodes is unclear, theories include neuron‐astrocyte uncoupling and microvascular disease with endothelial injury from defective oxidative phosphorylation.^38^, ^165^ Headaches in MELAS are theorized to be multifactorial, arising from energy failure, neuronal hyperexcitability, and vascular/endothelial dysfunction, rather than hypoperfusion.^166^, ^167^ Prevalence of headaches is as high as 70% in patients with MELAS^168^; however, evidence for specific treatments for headache in this population remains limited.

#### Triptans and ergots

Data is mixed on efficacy of triptans in MELAS‐associated headache. Subcutaneous sumatriptan was effective for headaches attributed to MELAS in two patients.^39^ Another case series reported efficacy of oral naratriptan and sumatriptan, while frovatriptan was ineffective in a patient with SSBP1 mitochondriopathy.^38^, ^87^ Whether this variability reflects differences in individual drug mechanisms or genotype‐specific responses remains unclear and warrants further study.

#### Anti‐hypertensive agents

In a recent survey, four of 34 patients with MELAS and chronic headache reported using metoprolol or propranolol, with partial benefit reported in two patients.^86^ Other studies noted limited headache relief with BBs due to dose‐limiting hypotension.^87^ The CCB flunarizine was also trialed and was well tolerated with initial benefit but showed decreasing efficacy over three years.^87^ In another patient, flunarizine was discontinued due to adverse effects.^38^ Taken together, there is some promise for use of anti‐hypertensive agents, but results have been mixed in this population.

#### CGRP class

Galcanezumab reduced headache frequency by >50% in a patient with SSBP1 mitochondriopathy with no reduction in effectiveness over time.^87^ Subcutaneous erenumab also led to significant reduction in headache frequency and severity in one patient with MELAS, but headache worsened upon cessation.^38^ The authors posit CGRP release improves microcirculation and may be responsible for migraine in MELAS. As many patients with this disorder are also treated with L‐arginine, a potent vasodilator, CGRP blockade may blunt this effect.

### SWS

SWS is a somatic *GNAQ*‐related neurocutaneous disorder affecting the eye (glaucoma), skin (capillary malformations), and brain (e.g. posterior leptomeningeal angiomatosis, venous malformations, parenchymal calcification, developmental venous anomalies).^169^ Neurologic risks include epilepsy, focal deficits, stroke‐like episodes, intellectual impairment, developmental delay, psychiatric disturbance, and migraine.^169^ Some patients are presymptomatically treated with antiseizure medications and aspirin.^170^, ^171^ Headaches affect up to 71% of individuals with SWS,^172^ often starting in childhood and in association with neurologic deficits in up to 58%.^173^ Prolonged hemiplegia without ictal correlate or infarct is reported in up to 30% of cases, and hemiplegic attacks may also occur following head trauma.^174^, ^175^ Prolonged visual aura or hemianopsia without parenchymal infarction has also been observed.^176^, ^177^

#### Triptans and ergots

Triptans were self‐reported as more effective than over‐the‐counter analgesics (42% vs. 20%) but caused worsening symptoms and/or focal deficits in 13% of patients.^40^ Naratriptan has been used without adverse effects in a patient with SWS with a history of prior cerebral infarction.^41^

#### Anti‐hypertensive agents

Flunarizine has shown benefit in pediatric patients with SWS with migraine and vascular events, significantly reducing headache severity, frequency, and duration with good tolerability.^88^ Headache resolution occurred after the first month in five of 20 patients.^88^ Given altered flow mechanics through leptomeningeal angiomatosis, abrupt changes in blood pressure should be avoided.

#### CGRP agents

There is currently no direct evidence in the medical literature regarding the role of CGRP agents in treating headache associated with SWS. There may be a theoretical risk in limiting compensatory vasodilation after infarction, but more data is necessary to explore this association.

#### Other agents

Data on NSAIDs are limited. Ibuprofen is one of the most common acute treatments for headaches associated with SWS based on patient self‐report.^40^ Caution must be exercised when considering NSAIDs if taken in addition to daily aspirin, which is used frequently in patients with SWS with the goal of reducing thrombosis and promoting perfusion.

Among other medications used for headache in SWS, one case study reported efficacy of lamotrigine in a young adult.^41^ Other patient‐reported preventive agents include amitriptyline (which may lower seizure threshold), gabapentin, valproic acid (VPA), and topiramate, though the latter should be avoided due to glaucoma risk.^40^

## ACQUIRED OR PROGRESSIVE VASCULOPATHIES

### MM

MM is a rare, progressive steno‐occlusive neurovascular disorder marked by stenosis of the terminal internal carotid artery (ICA) and proximal MCA and/or anterior cerebral artery (ACA). The subsequent development of prominent small‐vessel feeding collaterals and aneurysms can result in stroke or intracranial hemorrhage.^178^, ^179^, ^180^ Systemic vessels (e.g. carotid, renal, pulmonary, coronary) may be involved.^181^ Moyamoya disease (MMD) refers to idiopathic cases, whereas Moyamoya syndrome (MMS) is associated with other underlying medical conditions.^179^, ^180^ Management typically includes surgical revascularization and aspirin in non‐hemorrhagic cases.^179^ Headache occurs in up to 50% of patients and is hypothesized to originate from chronic hypoperfusion causing sensitization of brain tissue.^43^, ^182^, ^183^, ^184^

#### Triptans and ergots

Triptans and ergots should generally be avoided in patients with MM due to potential vasoconstriction in progressively stenotic major arteries relying on leptomeningeal collateral vessels.^44^, ^45^, ^46^

#### Anti‐hypertensive agents

Anti‐hypertensive medications are generally contraindicated in patients with MM to avoid watershed infarction.^90^, ^91^ CCBs, including verapamil and nicardipine, have shown benefit in MM headache by improving cerebral hemodynamics, though hypotension, decreased cerebral perfusion, and over‐distention of diseased vessels all remain concerns.^90^, ^91^ One study found that utilization of anti‐hypertensive agents to maintain normotension was associated with favorable outcomes.^89^ In patients with hypertension, cautious use of BBs and CCBs may be effective in managing headache.^89^

#### CGRP class

Although not formally contraindicated by the United States Food and Drug Administration (FDA) in this population, CGRP antagonists are also generally avoided in patients with MM due to impairment of compensatory vasodilation. In a mouse model of MCA occlusion, antagonism with olcegepant significantly impaired collateral flow and reperfusion success, leading to increased infarct size.^29^

#### Other agents

Enhanced platelet aggregation has been linked to headache onset in patients with MM, and aspirin may reduce headache risk by suppressing collagen‐induced aggregation.^43^ In a trial of 35 patients, all had reduced headache severity and frequency with aspirin, with minimal adverse reactions.^43^

### RCVS

RCVS is a cerebrovascular syndrome characterized by a beaded appearance of vessels and recurrent thunderclap headache accompanied by focal neurologic deficits.^47^, ^185^ RCVS is strongly associated with headache; up to 25% have a history of migraine, and up to 50% experience persistent headache post‐resolution.^49^, ^186^ Over 5% of cases recur, with complications including infarction, hemorrhage, and posterior reversible encephalopathy syndrome (PRES).^47^, ^49^

#### Triptans and ergots

Given their vasosensitivity and association with drug‐induced RCVS, triptans, ergots, selective serotonin reuptake inhibitors, SNRIs, and sympathomimetics should be avoided.^47^, ^49^, ^50^, ^51^, ^52^, ^53^ While triptans have been used in select cases, efficacy is inconsistent.^54^

#### Anti‐hypertensive agents

Treatment for RCVS acutely includes CCBs and magnesium.^92^, ^93^, ^186^, ^187^, ^188^, ^189^ Verapamil is used frequently for acute headache relief due to its vasodilatory qualities.^92^ Other effective CCBs include lomerizine, nimodipine, nicardipine for both acute and preventive management.^48^, ^54^, ^93^, ^94^ Caution must be exercised when using blood pressure‐altering medications to avoid watershed infarction^92^; however, propranolol has shown benefit in bath‐related headache (a subtype of RCVS), likely by mitigating sympathetic overactivity.^54^, ^92^ Gradual tapering is recommended when discontinuing CCBs, as one trial found that abrupt withdrawal of nifedipine resulted in RCVS recurrence.^94^

#### CGRP class

Due to their impairment of compensatory vasodilation, CGRP medications are generally avoided in the treatment of RCVS. Until more data are available, long‐term (>1 year) or lifelong avoidance following an episode of RCVS is recommended.^48^

#### Other agents

Glucocorticoids may worsen outcomes following RCVS, and as such should be avoided.^55^ Other preventive agents such as topiramate, VPA, and gabapentin may be effective in this patient population, but data is limited.^54^

### Other vascular stenosis/insufficiency

Vertebrobasilar insufficiency (VBI), defined as ischemia related to transitory hypoperfusion of the vertebrobasilar system, presents with symptoms such as vertigo, imbalance/ataxia, diplopia, visual loss, oropharyngeal dysfunction, and bilateral lower extremity weakness or drop attacks.^190^ VBI has also been associated with headache.^191^

#### Triptans and ergots

Triptans and DHE have been used without adverse effects in animal models and retrospective analyses of patients with VBI^56^, ^57^; however, extreme caution must be exercised in confirmed VBI given the risk of precipitation of acute watershed infarction of the posterior circulation.^58^, ^59^, ^61^

#### Anti‐hypertensive agents

Vasoactive medications should be used with caution in patients with VBI due to the risk of stroke and hypoperfusion, like considerations made in other steno‐occlusive conditions.^96^ In a trial of anti‐hypertensive agents in patients with intracranial stenosis, treatment with losartan and hydrochlorothiazide caused no adverse effects on cortical blood flow or cerebrovascular reactivity.^95^

## DISCUSSION

This narrative review was developed as a practical guide for clinicians who encounter pediatric patients with headache following stroke or in the setting of known or suspected cerebrovascular lesions. These patients present unique management challenges that require integrating knowledge of vascular pathophysiology with principles of headache medicine. Many therapies commonly used in primary headache disorders may pose risk when collateral‐dependent cerebral circulation, endothelial dysfunction, or altered autoregulation are present. By organizing the literature according to vascular etiology and summarizing the available evidence on both acute and preventive therapies, this review aims to provide clinicians with a framework for safe and individualized decision‐making (see Tables 1 and 2). We underscore the importance of assessing not only the mechanism of the headache but also the hemodynamic context in which it arises.

A central concern in managing these patients is the potential for harm from otherwise effective headache medications. Vasoconstrictive agents such as triptans and ergots, long regarded as first‐line therapies for migraine, carry theoretical and occasionally documented risks of ischemia or hemorrhage in patients with prior cerebrovascular injury and/or ongoing cerebrovascular abnormalities. Similarly, CGRP–targeting therapies, though often not formally contraindicated, may impair compensatory vasodilation in flow‐dependent lesions and warrant caution until more data are available. Conversely, certain anti‐hypertensive classes—including β‐blockers, CCBs, and ARBs—may offer both safety and neuroprotective benefit, particularly when hypertension, vascular or endothelial dysregulation coexist. Agents with minimal vascular impact, such as antiseizure medications, antidepressants, melatonin, and behavioral interventions, are appealing alternatives, though most supporting data derive from indirect or small studies.

Nonpharmacologic interventions, including both neuromodulatory devices and behavioral therapies, represent promising adjuncts or primary therapies for patients with vascular‐associated headache disorders. Devices such as external trigeminal nerve stimulators, noninvasive vagus nerve stimulators, and single‐pulse transcranial magnetic stimulators may provide acute and preventive benefit without interfering with cerebrovascular tone or hemodynamics. Similarly, biobehavioral therapies—including cognitive behavioral therapy, biofeedback, relaxation and mindfulness‐based approaches, and acceptance and commitment therapy—offer non‐vasoactive strategies that may improve coping and symptom control. Evaluation of these modalities was beyond the scope of this narrative review; however, both the American Headache Society^192^ and the International Headache Society^193^ have endorsed select neuromodulatory devices as reasonable options for acute and preventive treatment, albeit with varying strength of recommendation. Given their favorable safety profiles and minimal vascular impact, these approaches merit further investigation in patients with stroke‐related or vasculopathic headache syndromes, who may represent an ideal cohort for broader clinical evaluation and guideline refinement.

Despite increasing recognition of post‐stroke and vascular‐associated headache syndromes, these presentations remain relatively rare and under‐characterized. The scarcity of prospective studies, heterogeneity of underlying lesions, and limited documentation of medication exposure and outcomes impede precise risk–benefit assessment. In many clinical scenarios, decisions regarding headache management must be made despite limited or low‐quality evidence; in these cases, we recommend careful evaluation of the underlying cerebrovascular lesion and selection of therapies without clear physiological contraindications, with close monitoring and prompt discontinuation if headache frequency or severity worsens.

Additional limitations to this narrative review include the scarcity of pediatric‐specific studies evaluating headache therapies in the context of cerebrovascular disease, resulting in many management considerations being extrapolated from adult populations rather than derived from pediatric clinical trials. Additionally, as with all narrative reviews, the potential for publication bias must be considered, as studies demonstrating positive therapeutic effects are more likely to be published than those with neutral or negative findings. Future research should prioritize standardized phenotyping of vascular‐associated headache, longitudinal tracking of therapeutic interventions, and integration of imaging and physiologic biomarkers to clarify mechanisms of pain generation. Nuanced, multidisciplinary investigation will be critical to define evidence‐based treatment strategies that balance efficacy with cerebrovascular safety in this vulnerable population.

## AUTHOR CONTRIBUTIONS

**Allison C. Hyland:** Conceptualization; investigation; writing – original draft; supervision; writing – review and editing; project administration. **Samuel J. Belfer:** Conceptualization; investigation; writing – review and editing; project administration. **Christine K. Fox:** Writing – original draft; conceptualization; investigation; writing – review and editing. **Samantha L. Irwin:** Conceptualization; investigation; writing – original draft; writing – review and editing. **Rachel Vassar:** Conceptualization; investigation; writing – original draft; writing – review and editing. **Ann Oh:** Conceptualization; investigation; writing – original draft; writing – review and editing; supervision. **Sruthi Ilangovan:** Conceptualization; investigation; writing – original draft; writing – review and editing. **Amy A. Gelfand:** Conceptualization; investigation; writing – original draft; supervision; writing – review and editing.

## FUNDING INFORMATION

The authors declare that this research received no external funding.

## CONFLICT OF INTEREST STATEMENT

**Samantha L. Irwin** is a site PI on clinical trials investigating Ubrogepant and Atogepant in pediatric migraine. She is a site subinvestigator studying Rimegepant in pediatric patients with migraine. She has received an honorarium from The Hospital for Sick Children for speaking at Grand Rounds (2024) and travel support for the AHS REACH program (2024). In the last 24 months, she has received consulting income from Theranica, Pfizer, and Merz. Dr. Irwin is also on the editorial board for the Annals of The Child Neurology Society and Pediatric Neurology, for which she receives no income. In the last 24 months, **Amy A. Gelfand** has received royalties from UpToDate (for authorship), and honoraria from Elsevier (for authorship), the American Academy of Neurology (for editing) and the Weill Cornell Neurology Department and Kobenhavns Unversitet (for speaking). She receives a stipend from the American Headache Society for her role as Editor of Headache. She receives grant support from PCORI as a member of the Steering Committee for the REACH study and from the UCSF Resource Allocation Program as an investigator. She is also supported by a generous philanthropic donation made by Nathalie and Nicolas Giauque to the UCSF Child & Adolescent Headache Program. **Allison C. Hyland**, **Samuel J. Belfer**, **Christine K. Fox**, **Rachel Vassar**, **Ann Oh**, and **Sruthi Ilangovan** have no conflicts of interest to report.
